# The Patient-Provider Relationship Is Associated with Hepatitis C Treatment Eligibility: A Prospective Mixed-Methods Cohort Study

**DOI:** 10.1371/journal.pone.0148596

**Published:** 2016-02-22

**Authors:** Shari S. Rogal, Robert M. Arnold, Michael Chapko, Barbara V. Hanusa, Ada Youk, Galen E. Switzer, Mary Ann Sevick, Nichole K. Bayliss, Carolyn L. Zook, Alexis Chidi, David S. Obrosky, Susan L. Zickmund

**Affiliations:** 1 Center for Health Equity Research and Promotion, Veteran’s Administration, Pittsburgh Healthcare Service, Pittsburgh, Pennsylvania, United States of America; 2 Department of Surgery, University of Pittsburgh, Pittsburgh, Pennsylvania, United States of America; 3 Division of General Internal Medicine, University of Pittsburgh, Pittsburgh, Pennsylvania, United States of America; 4 Seattle Center of Innovation for Veteran-Centered and Value-Driven Care, Veteran’s Administration Healthcare Service, Seattle, Washington, United States of America; 5 Department of Biostatistics, University of Pittsburgh, Pittsburgh, Pennsylvania, United States of America; 6 Department of Population Health, New York University School of Medicine, New York, New York, United States of America; 7 School of Medicine, Oregon Health Sciences University, Portland, Oregon, United States of America; Harvard Medical School, UNITED STATES

## Abstract

Hepatitis C virus (HCV) treatment has the potential to cure the leading cause of cirrhosis and hepatocellular carcinoma. However, only those deemed eligible for treatment have the possibility of this cure. Therefore, understanding the determinants of HCV treatment eligibility is critical. Given that effective communication with and trust in healthcare providers significantly influences treatment eligibility decisions in other diseases, we aimed to understand patient-provider interactions in the HCV treatment eligibility process. This prospective cohort study was conducted in the VA Pittsburgh Healthcare System. Patients were recruited after referral for gastroenterology consultation for HCV treatment with interferon and ribavirin. Consented patients completed semi-structured interviews and validated measures of depression, substance and alcohol use, and HCV knowledge. Two coders analyzed the semi-structured interviews. Factors associated with patient eligibility for interferon-based therapy were assessed using multivariate logistic regression. Of 339 subjects included in this analysis, only 56 (16.5%) were deemed eligible for HCV therapy by gastroenterology (GI) providers. In the multivariate logistic regression, patients who were older (OR = 0.96, 95%CI = 0.92–0.99, p = .049), reported concerns about the GI provider (OR = 0.40, 95%CI = 0.10–0.87, p = 0.02) and had depression symptoms (OR = 0.32, 95%CI = 0.17–0.63, p = 0.001) were less likely to be eligible. Patients described barriers that included feeling stigmatized and poor provider interpersonal or communication skills. In conclusion, we found that patients’ perceptions of the relationship with their GI providers were associated with treatment eligibility. Establishing trust and effective communication channels between patients and providers may lower barriers to potential HCV cure.

## Introduction

Hepatitis C virus (HCV) affects 4 million people and is the leading cause of cirrhosis in the United States [1]. Despite increasingly efficacious treatments for HCV, less than 20% of infected patients have received HCV treatment [2,3]. Financial, logistical, and medical problems as well as patient-specific concerns all contribute to these low treatment rates. While the shift to interferon-free therapies has simplified the assessment of treatment eligibility, this assessment requires HCV diagnosis and referral for specialty care. Despite systematic efforts to increase rates of disease recognition and subsequent referrals to specialty clinics [4], up to 66% of referred patients do not attend the scheduled consultation for HCV therapy [5]. For those who do attend their appointments with specialized providers, potentially important barriers must be overcome before a patient is deemed eligible for treatment. The determination of treatment eligibility involves medical testing to define liver disease stage and relevant comorbid conditions, and the need for this workup has been associated with decreases in the number of patients enrolling into treatment programs [5].

Beyond tangible medical criteria, guidelines established by the American Association for the Study of Liver Diseases also address less well defined criteria for treatment eligibility, including adherence [6]. This requirement for eligibility is based on the need for consistent drug exposure in order to achieve a sustained viral response (SVR), which may become increasingly important with the high costs of the more recently introduced antiviral agents at a time of limited resources. The true impact of adherence and other non-medical eligibility criteria on treatment rates have not been fully explored, but may contribute to the significant variability in treatment eligibility, which ranges from 4 to 50%[7–9].

Providers continue to determine whether patients are eligible for HCV treatment, even in the era of interferon-free treatments. Given that the medical contraindications to interferon-free HCV treatment are far less, the non-medical criteria for eligibility, such as perceived adherence, are likely to become increasingly important. The aim of this study was to evaluate the determinants of provider-determined eligibility for HCV treatment with a focus on the patient perspective.

## Patients and Methods

### Subjects

**Fig 1:**
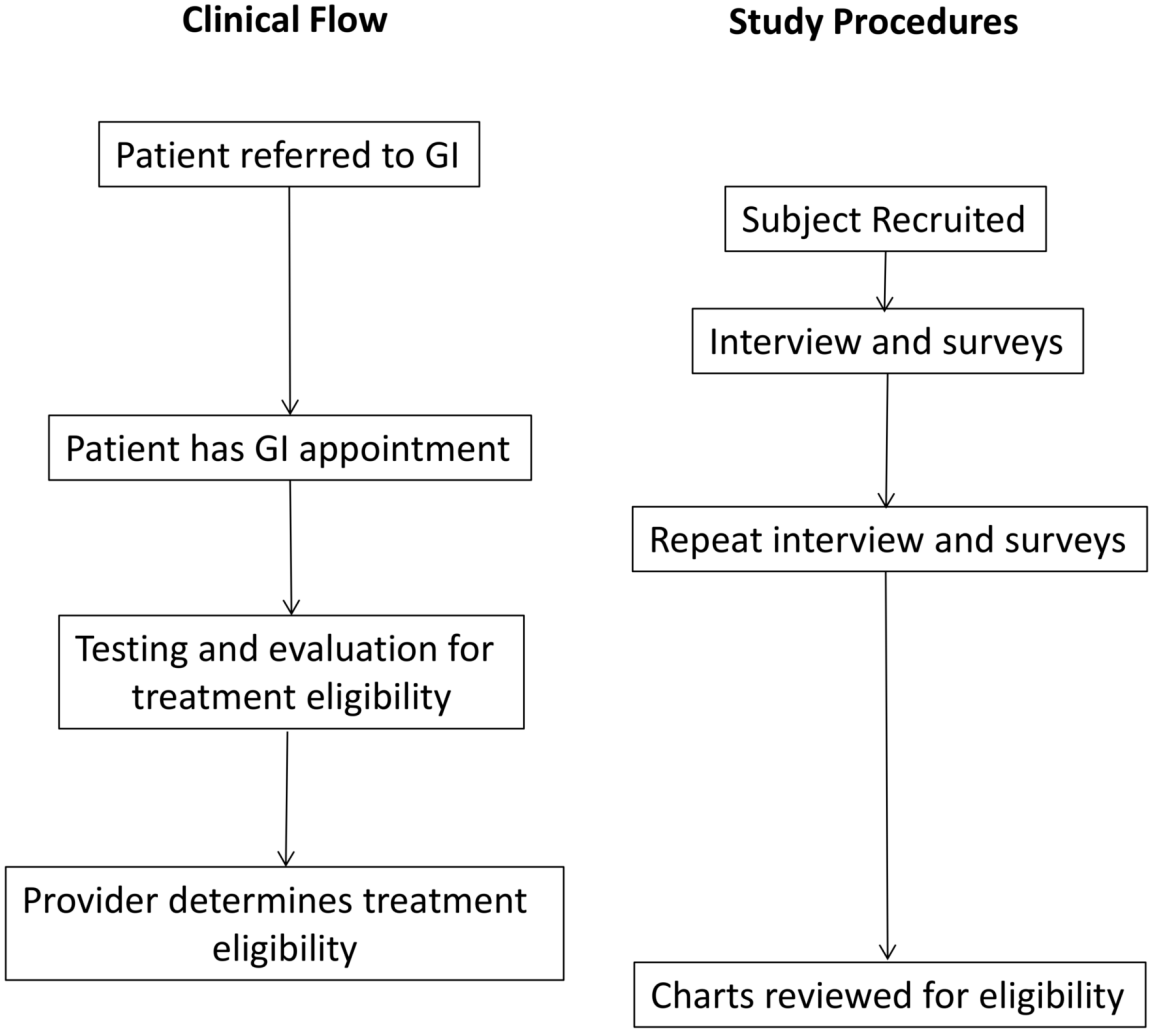
Clinical and Study Flow.

The VA Pittsburgh IRB approved this study with a waiver of HIPAA and a waiver of written informed consent. Interested patients were orally consented by research staff who then documented this oral consent in the research records. Subjects were considered eligible if they were referred to gastroenterology/hepatology (GI) providers to consider HCV treatment. We mailed invitations to each potential subject and followed up with a second invitation if there was no response within 1 week. Patients who still had not responded were invited to participate by clinic staff when they were called with routine appointment reminders. A study coordinator contacted all potential participants who expressed interest to obtain informed consent and complete an interview before and after their gastroenterology appointments. After the initial appointment with GI, all participants were asked to provide feedback about their experience, the information they learned, and their current attitudes toward treatment (S1 File). These interviews were approximately one hour and conducted over the telephone. Interviews were conducted prior to patients knowing their eligibility status, which was determined after follow-up. The flow of patients through the clinical and research procedures is outlined in Fig 1. Subsequent to the interview, patient records were abstracted to determine treatment eligibility. This protocol was approved by the VA Pittsburgh Institutional Ethics Review Board.

### Determination of Treatment Eligibility

Charts of the participants were systematically reviewed by members of the research team from the initial visit to 18 months or until patients began therapy, whichever came first. The notes by the GI providers were evaluated to determine treatment eligibility as a dichotomous outcome. Participants were deemed eligible if the provider explicitly confirmed that the participant was an appropriate candidate for therapy and/or should move toward treatment. In the absence of this documentation by the pre-determined study end-point of 18 months, participants were operationally deemed ineligible for the purposes of this study.

### Semi-structured Interview

We conducted semi-structured interviews after the consultation appointment (please see S1 File for the interview script). We digitally recorded each interview and used a “quasi-statistical” qualitative methodology developed by Crabtree and Miller to identify qualitative codes and to determine their frequency [10]. Through an iterative process, we constructed a qualitative codebook designed to capture patient-expressed barriers and facilitators to HCV treatment. The senior author and the master coder (SLZ and NKB) led teams of two trained coders as they analyzed audio files and abstracted qualitative data. Coders met to reconcile differences until 100% agreement was reached. The adjudicated codes were then added into a final databank that was then used for all subsequent analyses.

### Qualitative domains

We coded each potential barrier or facilitator to treatment initiation that participants discussed. The iterative review for the codebook construction was used to generate a list of themes and functioned as the framework for the coding of all the interviews. Based on this framework, we coded five main barrier and facilitator domains: physical symptoms and quality of life, access and coordination of care, provider-patient communication, personal barriers/facilitators, and social barriers/facilitators. All qualitative themes were coded as dichotomous (not mentioned = 0, yes mentioned = 1)

### Questionnaires

Patients provided demographic information and completed a telephone interview that included 5 validated quantitative measures. The Patient Education about Hepatitis C (PEAHC) collects baseline knowledge about HCV, its complications, risk factors, behaviors that exacerbate liver damage, current treatment options, treatment side effects, and treatment effectiveness rates [11–13]. It is scored by counting the number of items answered correctly. The Center for Epidemiologic Studies-Depression (CES-D), a widely used screening measure for assessing the presence and severity of depression, was used because it has been validated in HCV [14]. It uses 4-point Likert scales with item scores ranging from 0 = "rarely" to 3 = "most of days," with a score of 16 representing potentially relevant depression. In a screening of HCV patients CES-D had a Cronbach’s alpha between 0.88–0.90 when used on pre- and post-treatment patients [14]. The Alcohol Use Disorders Identification Test (AUDIT) was employed to identify hazardous drinkers [15]. It uses a 5-point scale from 0 = “never” to 5 = “daily use” with a score < 8 indicates non-hazardous alcohol use, 8–14 indicating hazardous, and >14 as dependent drinking. A review of research on the AUDIT found that the scale generally has a Cronbach's alpha reported to be in the 0.80's [16]. The Drug Abuse Screening Test 10 (DAST 10), a 10-item questionnaire, assesses drug use during the preceding 12 months [17]. It has a Cronbach’s alpha of 0.87 and uses 5-point Likert scales ranging from never to daily use [18].

### Statistical Methods

Descriptive statistics (means and standard deviations for continuous variables and frequencies for categorical variables) were computed to describe the sample. For the demographic and survey variables, we compared those who were eligible for treatment to those who were not using Student’s t tests (or Mann-Whitney) for continuous data and Chi-Square tests (or Fisher’s exact) for categorical data, to identify potentially important statistical covariates of treatment eligibility. For the qualitative variables, sets of the qualitative variables were aggregated into domains and coded as mentioned vs. not mentioned. We used logistic regression to identify the important qualitative predictors of treatment eligibility first with specific variables within a domain and then with domains themselves. To handle non-identifiability and multicollinearity that arose when specific qualitative variables within domains were sparse (<5 responses per item), we dropped the sparse specific qualitative variables and only included the larger domains.

Potentially relevant variables were identified based on p<0.15 in the univariate analyses and were included in multivariable logistic regression models that combined qualitative and quantitative data. Backwards elimination was then used to find a parsimonious final model. Multicollinearity was assessed by computing variance inflation factors (VIFs) for the full multivariate model prior to the final backwards elimination. VIF statistics >10 indicate the presence of collinearity. Stata (release 12; 2011, College Station, TX, StataCorp, LP) was used in all analyses.

## Results

### Participants

Six hundred and six patients were referred to GI to discuss the initiation of HCV antiviral therapy between 2006 and 2010 and were eligible for the PATHS study. Of the 477 subjects consented to be in the study, 39 could not be reached and an additional 76 people were excluded as their interviews were incomplete due to technical problems with the audio recorders. Of the 362 original PATHS study participants, an additional 23 people were excluded as they did not complete the semi-structured interview given after the initial GI consult, resulting in 339 (50.1%) study subjects in this analysis. The inter-coder reliability kappa statistic was 0.66–1.00, based on double coding 25% of the interviews. As shown in Table 1, participants were 98% male, which is in keeping with the demographics of the VA, 54% White and had a mean age of 54. The majority of the group had low annual incomes and low levels of education. Only 25% of the subjects were working, and over 36% lived with a spouse/partner. Based on the survey data, 34% of the cohort met criteria for hazardous or dependent alcohol use, 55% occasionally or consistently took illicit drugs, and 47% met the definition of depression based on the CES-D score of ≥16.

**Table 1:** Demographic and Clinical Characteristics.

Characteristic	Study Sample (n = 339)
**Age (mean±sd)**	54.2±6.9
**Comorbidity Index (mean±sd)**	1.1±1.7
**Gender** **N(%)**	
**Male**	332 (97.9)
**Female**	7 (2.1)
**Race** **N(%)**	
**White**	184 (54.3)
**Black**	153 (45.1)
**Other**	2 (0.6)
**Education** **N(%)**	
**High School or Less**	174 (51.3)
**Some Education after High School**	123 (36.3)
**At Least 4 Year College Degree**	40 (11.8)
**Income** **N(%)**	
**<10K**	123 (36.3)
**10–19K**	123 (36.3)
**20K+**	85 (25.1)
**Living Arrangement** **N(%)**	
**Alone**	125 (36.9)
**With Spouse/Partner**	125 (36.9)
**With Relatives**	46 (13.5)
**With Non-Relatives**	43 (12.7)
**Current Work Status** **N(%)**	
**Currently Working**	86 (25.4)
**Disabled or Retired**	168 (49.5)
**Not Working**	78 (23.0)
**AUDIT–C** **N(%)**	
**Non-Hazardous Alcohol Use**	223 (65.8)
**Hazardous Alcohol Use**	47 (13.9)
**Dependent Alcohol Use**	68 (20.0)
**DAST** **N(%)**	
**Non-Use**	150 (44.3)
**Occasional Use**	76 (22.4)
**Consistent Use**	112 (33.0)
**CES-D (Total) (mean ±sd)**	16.9±12.7
**CES-D** **N(%)**	
**<16**	180 (53.1)
**16+**	159 (46.9)
**PEAHC** **(mean±sd****)** **Post appointment % correct**	83.2 ±7.0

### Univariate Analysis

Fifty-four (16%) of the 339 participants were deemed eligible for treatment within 18 months of their index appointment. The factors associated with treatment eligibility in the univariate analysis are shown in Table 2. Those who were deemed eligible were significantly more likely to live with a partner. Other factors that had a borderline association with eligibility were lower levels of depressive symptoms, knowledge of HCV, and employment. For the qualitative data, several identified themes were associated with higher or lower likelihood of treatment eligibility. Participants were more likely to be deemed eligible for therapy if they described treatment in terms of improving their quality of life. Conversely, subjects who reported barriers related to the relationship with the GI provider were significantly less likely to become eligible for treatment. Additionally, the qualitative domain “patient barriers”—defined by expressed lack of interest in treatment, trouble with decision making, and/or financial or travel concerns—was significantly associated with decreased treatment eligibility (p = 0.045) in univariate analysis.

**Table 2:** Variables Meeting Selection Criteria from the Univariate Analyses of Treatment Eligibility.

	Eligible (n = 54)	Not Eligible (n = 285)	p-value^a^.
**Living Arrangement N (%)**			0.022
**Alone**	12 (22.2)	113 (39.6)	
**With Spouse/Partner**	23 (42.6)	102 (35.8)	
**With Relatives**	13 (24.1)	33 (11.6)	
**With Non-Relatives**	6 (11.1)	37 (13.0)	
**Work Status**			0.071
**Working or Retired**	38 (70.4)	163 (57.2)	
**Disabled**	16 (29.6)	122 (42.8)	
**CES-D Total Score**	13.6±10.3	17.5±13.0	0.054
**CES-D**			0.006
**<16**	38 (70.4)	142 (49.8)	
**16+**	16 (19.6)	143 (50.2)	
**PEAHC (****% Correct)**	85.0±5.8	82.9±7.2	0.067
**QUALITATIVE DATA**^b^.			
**Facilitator: Quality of Life**	46 (85.2)	192 (67.4)	0.017
**Facilitator: Patient: Decision Making**	4 (7.4)	8 (2.8)	0.097
**Barrier: Past Treatment Stopped by Provider**^c^	2 (3.7)	1 (0.4)	0.050
**Barrier: Provider-Patient Relationship**	9 (16.7)	92 (32.3)	0.025
**Barrier: Patient**	4 (7.4)	51 (17.9)	0.045
**Barrier: Social Work**	6 (11.1)	15 (5.3)	0.098

^a^ For continuous variables, p-value is based on Student’s t-tests (or Mann Whitney), for categorical variables, p-value based on chi-square tests (or Fisher’s Exact)

^b^ Shown is the number of times the facilitator or barrier was mentioned

^c^ Not included in the multivariable modeling model due to small numbers

### Multivariate analysis

The results of the multivariable model (Table 3) indicate that older participants (p = 0.049) and persons with depressive symptoms (p = 0.001) were significantly less likely to be eligible for treatment. In addition, barriers related to the relationship with the GI provider (p = 0.020) were associated with lower treatment eligibility. Multi-collinearity was not found for this model (all VIFs were <10).

**Table 3:** Multivariable Logistic Regression Model for Treatment Eligibility.

**Age**	0.96	0.92–0.99	0.049
**Barrier-Provider Patient Relationship**	0.40	0.10–0.87	0.020
**Depressive symptoms**	0.32	0.17–0.63	0.001

### Relationship with GI Provider: Detailed Thematic Analysis

Within the category coded as barriers related to the relationship with the GI provider, we examined specific themes that helped to clarify patients’ views. Themes were then extracted from the narrative data of the participants who were not deemed eligible to provide further insights into the barriers they experienced. First, participants discussed problems related to general interpersonal communication with the GI provider, focusing primarily on a feeling of personal connection. For example, when asked about whether the Veteran felt comfortable sharing needed information with the GI provider, one participant stated: “No. They weren't interested in anything I had to say.” Another described the provider as robotic or going through the clinical routine without emphasizing an interpersonal connection: “I don't know. I guess it just seems that [the provider] seemed to go through it by rote. I didn't feel much interest on [the provider’s] part as far as me as a person. It was more like [the provider] was going through a series of motions that [the provider has] done for so many times that…[it was] very mechanical.” Such experiences were also expressed in statements about a lack of interpersonal warmth. For example, when asked what negative experiences a patient had with their GI provider, one man stated: “I didn't really get the sense that they really cared about you as a patient, you know what I mean?” Consistent with the perceived problems in patient-provider communication, participants identified communication as an important area for improvement, such as shown in the following statement:

It's all about communication skills. You know, you can't just sit there and tell somebody: "Hey, gee, you've got hepatitis C and it kills you and all this," because [the providers] don't have it. So they have no feeling…they send you out of the office with a potential life-threatening disease. They don't care.

Secondly, participants noted that they had not received sufficient information from the provider in order to understand their disease or the treatment options. One patient, when asked whether there was additional information he would have liked to learn from his provider, added: “Well one thing I'd have liked to find out was: what was the treatment for hepatitis C? Find out if I was going to go through it? They wouldn't even give me that information.” Participants felt compelled to obtain information independently: “The only thing is that I don't have enough information on really what's going on. I've been going to the library to do my own research, and I don't know a whole lot of information on it.”

One additional important point emerged that related to communication skills, as participants preferred an open discussion with their providers rather than receiving hand-outs or pamphlets. Beyond the need for discussions that focus on individually relevant facts, participants also considered the available information as too complex or too confusing to guide their decisions:

No. I'm just at a loss right now. I can't quite help you much, except I got to say: try to educate people on what the hell's going on with them. You know, don't send them literature. Ask the people. My eyes are so shot to the level that I can't see no more. You know what I'm saying? And they give me a big pamphlet with all these wee little lines and I'm going to sit there with a magnifying glass and make my way through it? And then how much of it did I retain? How much of it did I gather? You need to have an explanatory class on this mess.

Finally, some participants felt stigmatized by healthcare providers. One man felt judged when he told his provider that he used Vicodin for pain, adding “but the way they try to pigeon-hole me and I'm a damn junkie or something, you know? That's what bothers me.” Another patient, who described himself as a moderate drinker, expressed his frustration with his GI provider:

[The provider] said, "Well, we have a program and if you are in the program you can't drink." I said, "Well, that's fine." And then says, "Well, I tell you, I'll give you the information, but we can't do anything for you until for 6 months until you stop drinking." You know, like "what?" And [the provider] says to me that [they] believe I drank more than that. It's really pissing me off at the time. “Well, wait a minute, you know. Yeah, I do drink on occasion and I know how much I consume because … I don't drink at home.” I said, "And if I do drink at home it's because I went out to dinner with my wife and I'm the designated driver and so I don't drink and drive." "Yeah right," you know, was [the provider’s] comment. So, I'm already into this really pissed off place, you know?

Such responses were also expressed by patients who described themselves as former or active drug users: “If you are a drug addict or used to be a drug addict, they look down upon you…I don't think it's right, you know, because I had tracks on my arm. They look at you like you are *eewh*! You know?” Nonverbal signals similarly conveyed a sense of stigmatization. Such perceptions primarily focused on the standard precautions that are routinely implemented when handling potentially infectious biological materials, as shown in the following statement: “They—you're in the hospital and they act like you have leprosy, you know. They put signs up, you know, about communicable disease or something like on that order, you know. That gets pretty frustrating.”

## Discussion

Patients describing more positive relationships with their hepatology providers were more likely to be deemed eligible for HCV treatment. While other studies have demonstrated the importance of other factors in HCV treatment eligibility, this large, prospective, mixed-methods study adds to the literature by providing a rich contextual understanding of how the quality of patient-provider interactions may influence measurable health outcomes. The themes emerging from the qualitative analysis include the importance of communication and connection between patients and providers. Patients spoke of desiring more warmth from providers and wanting information in the form of conversation rather than pamphlets. Stigma also emerged as a major theme for patients, particularly among those with a history of prior drug use.

This large, mixed-methods cohort adds to a growing body of literature regarding HCV treatment eligibility. In this cohort, specialists found only 16% of participants to be eligible for interferon-based treatment; this is consistent with previously-published interferon-based treatment eligibility rates [19,20]. Prior studies assessing treatment eligibility have focused on traditional quantitative factors and not on qualitative factors. For example, a study of 4,084 Veterans found that 32% were eligible for treatment based on standard criteria and that non-eligibility was associated with ongoing substance use and medical and psychiatric comorbidities [21]. Among those deemed eligible, an additional 24% declined to be treated. Another study with a treatment eligibility rate of 16–26% using standard guidelines and that age was significantly associated with treatment eligibility as determined by a clinician [20], consistent with our findings. Similarly, another study found that non-treatment was associated with active substance abuse, appointment non-attendance, early-stage disease, and medical and psychiatric comorbidities [7]. Medical comorbidities, cytopenias, hepatocellular carcinoma, older age, and advanced liver disease were also associated with non-eligibility in yet another study [9]. While several studies have assessed medical factors associated with treatment ineligibility, there have not been large studies of qualitative factors associated with HCV treatment eligibility. This is notable because medical factors may be less relevant in the interferon-free treatment era. While these treatments dramatically decrease side effects and medical contraindications to therapy, the providers continue to determine patient eligibility for these costly treatment regimens. The qualitative factors associated with this determination are critical to understand in order to maximize treatment eligibility in a new era.

Existing literature supports the importance of the patient-provider relationship to health outcomes. Communication is a critical part of the patient-provider relationship. We have previously described that 40% of patients with HCV perceived communication problems with their treating clinician [22]. This poor communication on the part of the provider can lead to the patient perception of stigma [23–27]. Additionally, the overall quality of the patient-provider relationship has been associated with medication adherence [28]. The importance of patient-provider rapport has been demonstrated to be associated with improved HIV medication adherence [23,29] and with improved outcomes [30]. It is unclear from this study whether the patient-perceived relationship issues could be improved with changes in the provider’s communication skills. Given that communication skills are teachable [31], this should be assessed in future studies. Additionally patient-directed interventions may aid in rapport and relationship building. For example, patients may be empowered with more knowledge about the treatment process if the educational materials were derived with patient input.

In addition to the patient-provider relationship, age and depression were also independently associated with decreased eligibility for HCV therapies. Advanced age and severe depression were contraindications to HCV therapy at the time of this study, per national guidelines[32]. These contraindications were somewhat justified by studies of interferon-based regimens. Advanced age has been associated with lower sustained viral clearance rates in some [33,34] but not all [20] studies, and with increased overall comorbidity and typically a longer duration of HCV infection. These factors may translate to more advanced disease, making treatment more difficult. Similar to advanced age, depression was a relative contraindication to interferon-based treatment [35] and thus significantly correlated with a lower likelihood to move toward treatment eligibility [36,37] in other studies. Consistent with our findings, a prior study demonstrated that 40% of HCV infected patients with coexisting psychiatric diseases were not eligible because of the severity of mental health disorder and/or did not complete their evaluations [38]. Correspondingly, mental health treatment has been shown to improve HCV treatment eligibility [39,40]. Though depression is not a contraindication to interferon-free HCV treatment regimens, patients with psychiatric comorbidities have had higher rates of incomplete evaluations in the past [41], which may still affect decisions about using the newer, very expensive treatments in this population. Thus, the finding that depression and age were significantly associated with treatment ineligibility in this study is consistent with prior literature.

While the prospective design, large cohort, and rich data set provide important insight, this study has several limitations. Our results are, in part, derived from qualitative methods which limit generalizability of findings, but which impart an important richness in detail. From a methodological perspective, qualitative research relies on the open-ended response from each participant, which provides important in-depth information, but also requires rigorous coding. Our study used a semi-structured interview guide that asked questions in a specific and pre-determined order that helped to reduce inconsistent responses. The advantage of the open-ended approach is that, by incorporating the patients’ words, we can discover barriers and facilitators that were not previously considered and would not have been included in an *a priori* survey design. Additionally, we applied a rigorous approach with multiple raters and achieved high inter-coder reliability. In addition, the generalizability of findings may be limited by our focus on US Veterans’ treatment within a single VA Healthcare System. A VA sample is unique in that Veterans are provided complete healthcare and medications are at a near 100% subsidized rate. In private systems, insurance coverage may be a more relevant consideration in determining treatment eligibility, particularly in the context of more expensive interferon-free treatments. As such we were able to include the voice of low income patients who would likely be excluded in other healthcare systems due to limitations of insurance coverage. Also, this cohort included predominantly White and Black men but few members of other gender or racial/ethnic groups. Additional research will be needed to determine the extent to which the problems identified in this healthcare system are prevalent elsewhere. A final limitation of this study was the lack of data regarding disease characteristics or severity. These data were not collected at the time of the study as the IRB deemed such clinical data unnecessary to capturing patients’ perceived barriers to treatment given the qualitative goals of the study. The disease-level variables associated with treatment eligibility have been previously well-characterized [7,9,20,21,38,41]; this study adds to the existing data by providing a qualitative perspective. Despite these limitations, our qualitative and survey data provide a rich contextual understanding of the patient perspective regarding their relationship with providers, an understanding which will be critically important in the era of interferon-free treatment.

In conclusion, we found that patients’ perceptions of the relationship with their GI providers were associated with treatment eligibility. Establishing trust and effective communication channels between patients and providers may lower barriers to potential HCV cure. Future studies should assess how to optimize patient-provider rapport in order to improve treatment rates in the context of newer HCV treatments.

## Supporting Information

S1 File Patient Attitude Toward Hepatitis Study (PATHS) Interview Script Semi-Structured Interview Guide. (DOCX)
