# Supplementary material for: The Patient-Provider Relationship Is Associated with Hepatitis C Treatment Eligibility: A Prospective Mixed-Methods Cohort Study
Source: PLoS One. 2016 Feb 22;11(2):e0148596. doi: 10.1371/journal.pone.0148596 (PMC4763474; doi:10.1371/journal.pone.0148596)
Supplement: S1 File — (DOCX) [file pone.0148596.s001.docx]

**S1. Patient Attitude Toward Hepatitis Study (PATHS) Interview Script Semi-Structured Interview Guide**

*As you know we are interested in learning more about your perspective on having hepatitis C. In the next few minutes, I will ask you a series of questions. While you are doing this interview, please relate all of your answers to your last appointment that you had with the Gastroenterology provider regarding your hepatitis C. Please let me know if you did not understand any question. And remember, we want to hear your side of the story. So there are no right or wrong answers.*

(1) In general, what were your impressions of your first appointment with the provider at the Gastroenterology clinic?

(2) What did you learn from your provider about hepatitis C?

(3) What positive or negative experiences did you have with this provider?

(4) Is there anything that you learned that is particularly helpful for you?

(5) If any, what areas do you think Gastroenterology providers could improve upon when communicating with patients about their hepatitis C?

(6) Did the provider recommend that you move toward hepatitis C treatment?

**If so,** what is the next step the provider recommends you take?

(7) Are you interested in moving toward hepatitis C treatment at this time?

(8) What are your reasons in moving / not moving toward hepatitis C treatment?

(9) How important was the visit with the provider in helping you make up your mind on hepatitis C treatment?

(10) Did this visit change your views on hepatitis C treatment? **If so,** how?

(11) Did the provider tell you about your chances of clearing the virus based on your specific genotype or race?

(12) Do you feel that there are any roadblocks in moving towards treatment? **If so,** what are those?

(13) We are wondering how much trust do you place in this Gastroenterology provider?

1. If you could rate the trust you have in this provider between 1 (lowest) and 100 (highest) what rating would you give? Why?

(14) During your appointment, do you feel that you could connect and share needed information with your provider? **If not,** why?

(15) If you were to describe your level of knowledge of hepatitis C and its treatment from 1 to 100, what rating would you give yourself? What does this score mean to you?

(16) Was there any additional information that you would have liked to learn from your provider that you didn’t learn at this appointment?

(17) Is there anything you would like to add that would help us better understand your experience?
